# 3D transcranial ultrasound localization microscopy for discrimination between ischemic and hemorrhagic stroke in early phase

**DOI:** 10.1038/s41598-022-18025-x

**Published:** 2022-08-26

**Authors:** Arthur Chavignon, Vincent Hingot, Cyrille Orset, Denis Vivien, Olivier Couture

**Affiliations:** 1 Sorbonne Université, UMR 7371 CNRS, Inserm U1146, Laboratoire d’Imagerie Biomédicale, 15 Rue de l’Ecole de Médecine, 75006 Paris, France; 2 UNICAEN, Inserm U1237, Etablissement Français du Sang, Physiopathology and Imaging of Neurological Disorders (PhIND), GIP Cyceron, Institut Blood and Brain @ Caen-Normandie (BB@C), Normandie University, Caen, France; 3 Department of Clinical Research, Caen-Normandie University Hospital, CHU Caen, Avenue de la Côte de Nacre, Caen, France

**Keywords:** Diagnostic markers, Biomedical engineering, 3-D reconstruction, Ultrasound

## Abstract

Early diagnosis is a critical part of the emergency care of cerebral hemorrhages and ischemia. A rapid and accurate diagnosis of strokes reduces the delays to appropriate treatments and a better functional recovery. Currently, CTscan and MRI are the gold standards with constraints of accessibility, availability, and possibly some contraindications. The development of Ultrasound Localization Microscopy (ULM) has enabled new perspectives to conventional transcranial ultrasound imaging with increased sensitivity, penetration depth, and resolution. The possibility of volumetric imaging has increased the field-of-view and provided a more precise description of the microvascularisation. In this study, rats (n = 9) were subjected to thromboembolic ischemic stroke or intracerebral hemorrhages prior to volumetric ULM at the early phases after onsets. Although the volumetric ULM performed in the early phase of ischemic stroke revealed a large hypoperfused area in the cortical area of the occluded artery, it showed a more diffused hypoperfusion in the hemorrhagic model. Respective computations of a Microvascular Diffusion Index highlighted different patterns of perfusion loss during the first 24 h of these two strokes’ subtypes. Our study provides the first proof that this methodology should allow early discrimination between ischemic and hemorrhagic stroke with a potential toward diagnosis and monitoring in clinic.

## Introduction

Stroke is among the most serious health issues of the century with more than 13 million cases every year worldwide^1,2^. Most of them, 80%, are ischemic strokes that occur when the cerebral blood flow is dramatically reduced following clot-induced arterial occlusion, for example, depriving parts of the brain of oxygen and glucose^3^. The other 20% are hemorrhagic strokes with blood leaks into the brain parenchyma due to a rupture of a vessel, leading to both hypoperfusion and increased intracerebral pressure^4^. In both cases, non-supplied tissues die in a few hours and leave patients with serious cognitive impairments and high mortality rates. The care of ischemic stroke is rtPA-mediated fibrinolysis in a short therapeutic window of 4.5 h^5,6^, but recent studies suggest an extension up to 9 h^3^. When possible, the mechanical thrombectomy can be performed to remove the clot in situ in the first 24 h after stroke onset^7^. The treatments can be eventually combined with better functional outcomes and lower mortality^8^ in a therapeutic window of 6 h^9^. The treatment of hemorrhagic stroke consists of intracerebral blood removal and/or decompressive craniectomy^10,11^. In both cases, time is brain, with a necessity to make appropriate interventions as soon as possible. Also important, rt-PA treatment is associated with a risk of bleeding and thus should be prohibited on hemorrhagic stroke patients, in case of trauma or when anticoagulant treatments are recent^3^.

Thus, stroke management relies heavily on cerebral imaging techniques used to discriminate between ischemic and hemorrhagic stroke^12,13^ and thus preventing possible complications after treatments^14,15^. In particular, imaging is mandatory in the acute phase of the disease to address patients for fibrinolytics + /− thrombectomy treatments versus neurosurgeries^7,9,12^. It would also be needed at the subacute phase of the disease (24 h to 5 days) to monitor possible secondary cerebral hypoperfusion (ischemia), vasospasms or hemorrhagic transformations^16,17^. Although early brain imaging is performed thanks to CT or MRI to discriminate between ischemic and hemorrhagic strokes, post-stroke monitoring is insufficiently performed. Indeed, CT or MRI machines are either not possible at the bedside, usually over-booked, and more cost-expensive than echography.

Ultrasound imaging is currently not included in the clinical practices for stroke diagnosis. One main reason is that the human skull affects the propagation of acoustic waves and hinders the resolution and sensitivity for microvascular brain imaging. Nevertheless, the potential advantages of ultrasound imaging are multiple: repeatable, safe, bedside, affordable and mainly available, and it is already used to monitor major cerebral arteries with transcranial Doppler^18^. The introduction of Ultrasound Localization Microscopy (ULM)^19–23^ may partly solve the lack of sensitivity and resolution due to diffraction limit and skull attenuation thanks to intravascular microbubbles tracking. The better image quality enables imaging of the small vessels even behind the human skull and provides an accurate description of brain vascularization with hemodynamics^24,25^. Nonetheless, these first proof of concept are limited by the elevation projection and would require a plane-by-plane exploration with an experienced experimenter.

The elevation projection was solved with the emergence of volumetric imaging by combining thousands of transducers for 3DULM^26–29^. The system reaches a large field of view, an unbiased velocity estimation, and a quasi-isotropic resolution in-depth even though the intact skull bone^30^.

In this study, ischemic and hemorrhagic strokes were induced in rats and imaged in the early phases with 3DULM to describe perfusion losses in cerebral microvascularization. Cerebral ischemia was induced by a thromboembolic occlusion of the middle cerebral artery (MCA)^31–33^, and intracranial hemorrhage consisted of a collagenase injection in the striatum^34^. A microvascular diffusion index (MDI) was devised and evaluated at different stages and locations of the brain and demonstrated different patterns of perfusion losses between ischemic and hemorrhagic stroke’ models. We thus provide here evidence that 3DULM could be a relevant microvascular imaging modality to detect perfusion changes in the acute phase of stroke, particularly in the first hours of an MCA occlusion and thus to discriminate between ischemic and hemorrhagic stroke. This new device may diversify imaging tools for stroke triage, with better availability than MRI and CT scan, and an unprecedented sensitivity to intravascular flows.

## Methods

### Animal experiments

Animals’ experiments were performed under guidelines from the European Community Council (2010/63/EU) and approved by the protocol APAFIS #22544 validated by the French ethics committee “Comité d’éthique Normandie en matière d’expérimentation animale”. Reporting in the article follows the recommendations of the ARRIVE guidelines.

Sprague–Dawley male rats of 6–7 weeks old (290 ± 60 g) were used to validate the experiment, divided into two groups for ischemic model (n = 4) and hemorrhagic model (n = 5). Before this validation cohort, 11 animals were used to set the surgical and imaging procedures. All animals were anesthetized with a mix of isoflurane (2%) and nitrous oxide and installed in a stereotaxic frame. The temperature of the animals was regulated at 38 °C with a heating pad and a rectal probe.

**Figure 1:**
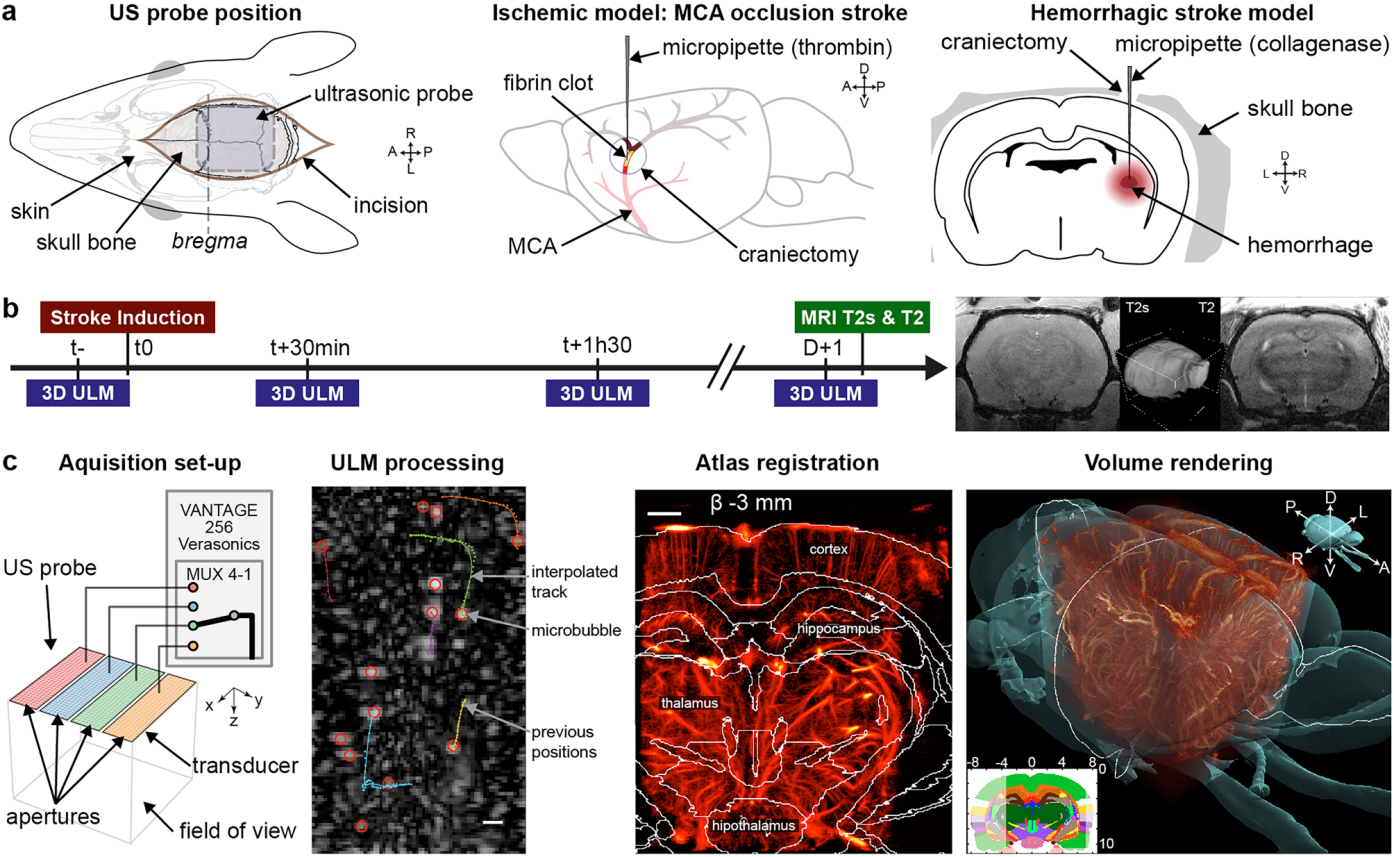
3DULM for in vivo angiography at the early phase of stroke’s models. **(a)** Skin opening for ultrasound imaging. Ischemic model: a small hole was drilled above the MCA branch, and human thrombin was injected. Hemorrhagic model: the parietal bone was drilled, and collagenase was injected in the striatum. (**b)** Timeline of the experiment for each model, with four acquisitions: in the half hour prior stroke onset (t−), in hyperacute stage (30 min and 1h30), and the day after with MRI imaging. T2* and T2 MRI sequences slices and 3D rendering. (**c)** Ultrasound matrix probe with transducers pooled in four synthetic apertures successively connected to the echograph. ULM processing with filtering, localization and tracking. Rat brain atlas registered on ULM volume (600 µm width slab). 3D rendering with brain surface with Amira software (Thermo Fisher). Scale bars: 1 mm. Figures were created with (**a–c**) Illustrator 2021 (https://www.adobe.com/products/illustrator), (**c**) MatLab 2019a (https://www.mathworks.com/products/matlab), (**c**) Amira 2019.4 (https://www.thermofisher.com/amira-avizo).

The top of the head was shaved, and the skin was incised for the stroke model’s induction and the convenience of ultrasound imaging (Fig. 1a). The skull was covered with echographic gel. The removal of the scalp did not affect the ULM results.

### Stroke models in the rat

The ischemic model consisted of an injection of human alpha thrombin (3 µl , 4 UI/µl) in the lumen of the MCA with a micropipette^31–33^ (Fig. 1a). The skin and muscle were incised between the right eye and ear. The temporal bone was drilled, and the dura was excised to access the branch of the MCA. The thrombin-induced a fibrin-rich clot, occulting the MCA.

The hemorrhage model was induced inside the striatum by injecting collagenase (2 µl) with a micropipette via a small hole drilled in through the parietal bone (Fig. 1a)^34,35^ (stereotaxic coordinates {AP: −1.3 mm, ML: 3.5 mm, DV: 4.5 mm}). Bleeding started at least 10 min later^34^.

Each model follows the same timeline with a baseline ULM imaging in the half-hour before stroke induction (t−) (Fig. 1b). Ischemic or hemorrhagic stroke models were then induced, and two ULM acquisitions were realized after 30 min and 1h30. The incision was sutured, and the animal was woken up. The day after, the animal was anesthetized again and imaged with the same position of the probe. No supplementary analgesics were used. Most animals were then scanned with a 7 T MRI (T2* and T2 weighted sequences, Bruker, USA) (Fig. 1b).

### 3DULM imaging

3DULM was extensively detailed in our previous paper^30^ including all specifications on the volumetric ultrasound sequence, the transcranial brain imaging on rat, and the ULM post-processing. Briefly, volumes were acquired on an ultrafast research scanner Vantage 256 (Verasonics, Kirkland, USA) with a 32 × 32 ultrasonic matrix probe (central frequency 7.8 MHz, 300 µm pitch). The 1024 elements were divided into 4 sub-apertures connected successively to the scanner via a multiplexer^36,37^ (Fig. 1c). Volumes were obtained with 5 compounded tilted plane waves (Fig. 1c). Each plane wave required 10 successive transmission-reception to collect all backscattered signals. For volumetric ULM imaging, 100 k volumes at 250 Hz compounded volume rate (CVR) were acquired in 7 min. 50 µl boluses of Sonovue microbubbles (Bracco, Italy) were injected in a tail vein catheter every 30 s during acquisitions. (Supplementary Materials A).

Data were beamformed with voxels of 150 × 150 × 99 µm^3^, and the tissue signal was rejected by removing the first 12 of 200 eigenvalues from the singular value decomposition^38,39^. Microbubbles were detected and then localized with a radial symmetry algorithm considering a full width at half maximum of 5 voxels in all directions^28,40^ (Fig. 1c). Microbubbles’ positions were paired into tracks using a Kuhn-Munkres based algorithm (*simpletracker*, Jean-Yves Tinenez) with a maximal linking distance of 0.3 mm and a minimum persistence of 37 ms. Tracks were smoothed with a moving average of 5 points, interpolated with a linear model, and binned in 9.9 µm wide cubic voxels yielding a 10.5 × 11.5 × 11.2 mm^3^ angiographic volume^30^. Almost 50 M microbubbles were detected in the 100k volumes and tracked into 500k trajectories. Tracks velocities were also averaged in each voxel of the volume. Volumes were manually registered with rigid transformations on a rat brain atlas^41^(Fig. 1c).

Signal and image processing were done with MatLab (The MathWorks Inc., release 2019a, MA, USA). 2D slices images renderings were also done with MatLab. All 3D renderings were performed with Amira software (Thermo Fisher Scientific Inc., release 2019.4, MA, USA).

### Microvascular diffusion index

**Figure 2:**
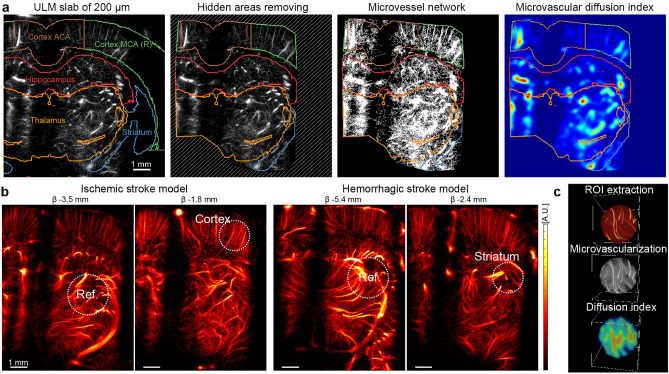
MDI for unsupervised observation reflects the vascularization density. **(a)** Anatomical regions are extracted from anatomical structures. The hidden areas are excluded from the study. MDI computation with a range of 100 µm on binarized vascularization. (**b)** Selection of a round reference region of 1.2 mm diameter in the thalamus and a region of interest in the cortex for ischemic stroke (0.9 mm diameter) and in the striatum for hemorrhage. (**c)** Example of a spherical ROI extracted from the ULM volume, binarization and MDI computation. Figures were created with (**a**,**b**) MatLab 2019a (https://www.mathworks.com/products/matlab), (**c**) Amira 2019.4 (https://www.thermofisher.com/amira-avizo).

A microvascular diffusion index (MDI) was devised from the 3DULM volume to quantify the density of microvessels. Microvessels can be associated with voxels where at least one microbubble has passed through. In theory, the voxel in a close neighbor to the passage of a microbubble (vessel) will be supplied with oxygen and nutrients more than voxels far from any trajectories. The quantification is expressed in a larger zone, exploiting the contribution of many trajectories to analyze the microvascular density at the scale of the lesion. However, this quantification still benefits from super-resolution since it is performed within the resolution of the initial volumetric ultrasound imaging. For each region of interest, it reflects the proximity of microvessels as a weighted sum of close microbubbles trajectories with a diffusion law. We hypothesize that dioxygen will diffuse within tissues on a distance of about 100 µm, as described in^42–44^. The microvascular network was first enhanced by binarizing the angiography volume (${Vol}_{Angio}$): each voxel with a least one microbubble was given a value of one, and in the absence of a microbubble, it was given a value of 0 (Fig. 2a). A Gaussian convolution filter was applied with a standard deviation of 100 µm (${R}_{{O}_{2}}$), representing approximately the diffusion range of dioxygen in the tissue by capillaries^42–44^.

$$MDI\left(\overrightarrow{x}\right)={\int }_{\Vert \overrightarrow{r}\Vert <{R}_{{O}_{2}}}{Vol}_{Angio}\left(\overrightarrow{x}+\overrightarrow{r}\right){e}^{-\frac{\Vert \overrightarrow{r}\Vert }{{R}_{{O}_{2}}}}.d\overrightarrow{r}$$

This value cannot be used as-is and was averaged on larger zones close to 1 mm^3^ to get an average value and a standard deviation of the microvascularization at a larger scale. Comparable indices have already been exploited, for example, in tumor vascularization with optical coherence angiography^45^.

### Unsupervised observation for MCA occlusion

For ischemic stroke, the MDI was computed in anatomical regions without prior knowledge of the stroke location. The regions were devised from the anatomical structures of the Waxholm Atlas. Five zones were analyzed: striatum, hippocampus, thalamus, and the cortex divided into two zones perfused by anterior cerebral artery (ACA) and MCA. For each region, the MDI values were normalized compared to the first timepoint before the stroke induction.

Sutures and lateral crests obstruct the propagation of ultrasound waves and hide a few regions that were excluded from the study to prevent any misinterpretation (Fig. 2a).

### Supervised observations

A specific analysis focuses on a particular region manually selected and adapted to each animal. It yields a characterization of an area with prior knowledge of the location of the lesion. Regions of interest (ROI) were defined for both stroke models to evaluate MDI (Fig. 2b). First, a 1.2 mm diameter spherical region was defined in the thalamus in a well-insonified region and far from the stroke influence zone (Fig. 2c). This region of interest was used as a reference for the normalization of volume: all volumes did not receive the same number of microbubbles and must be equalized. A second region was located inside the cortex for ischemic stroke or in the injection site for the hemorrhage model.

## Results

### Ischemic stroke model

**Figure 3:**
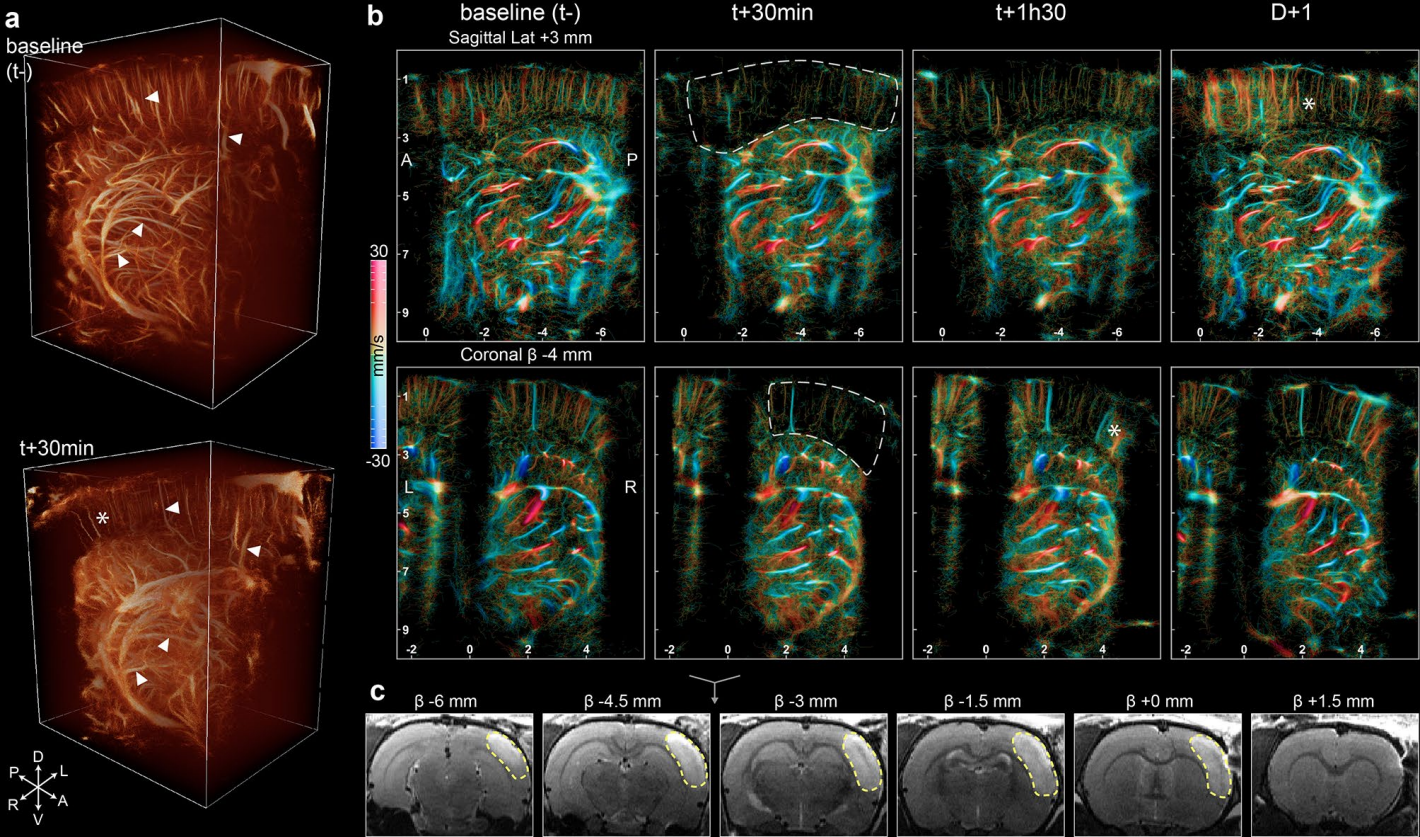
The MCA occlusion ischemic stroke was observed with 3DULM at the early phase. **(a)** 3D renderings at before and 30 min after stroke’s induction (Supplementary Video S1). (**b)** Sagittal and coronal slices of 600 µm with velocity rendering at different timepoints. Ischemic cortex wrapped with a white dotted line. ${V}_{max}=30\mathrm{ mm}/\mathrm{s}$. Upward flows: red, downward flows: blue. (**c)** T2 MRI at D + 1 with the segmentation of the lesion (yellow dotted lines). Scale bar: 2 mm. Figures were created with (**a**) Amira 2019.4 (https://www.thermofisher.com/amira-avizo), (**b**,**c**) MatLab 2019a (https://www.mathworks.com/products/matlab).

The injection of thrombin initiated a fibrin clot which obstructed the lumen of the proximal part of MCA. A hypoperfused region appeared in the downstream area visible in the 3DULM at 30 min (Fig. 3a, Supplementary Video S1). Large vessels could be identified in both volumes prior and 30 min after onset (white triangles), but the corresponding microvascularization was missing (white asterisk). Slices of 3DULM volumes allowed better identification of the ischemic regions. The hypoperfused area follows the cortex area (white dotted lines) (Fig. 3b). The blood velocity renders upward and downward flows visible with clear identifications of arteries and veins. The hypoperfused region drastically lacks microbubbles trajectories, confirming inexistent blood perfusion. Only a few wide blood vessels remained supplied. The monitoring of ischemia with volumetric ultrasound revealed the spontaneous reperfusion of the tissue in the next few hours after stroke onset. On this example animal, the microvascularization is slowly restored at t + 1h30 with sparse, isolated trajectories in the previously hypoperfused area (white asterisk). The day after, the reperfusion was more visible on the sagittal slice with the restoration of the initial complex mesh of microbubbles trajectories. The ischemic lesion was observed the day after with MRI with a T2 weighted sequence. Edema appeared hyperdense in a wide part of the right cortex and confirmed an extensive ischemic lesion of 90 mm^3^ (Fig. 3c).

**Figure 4:**
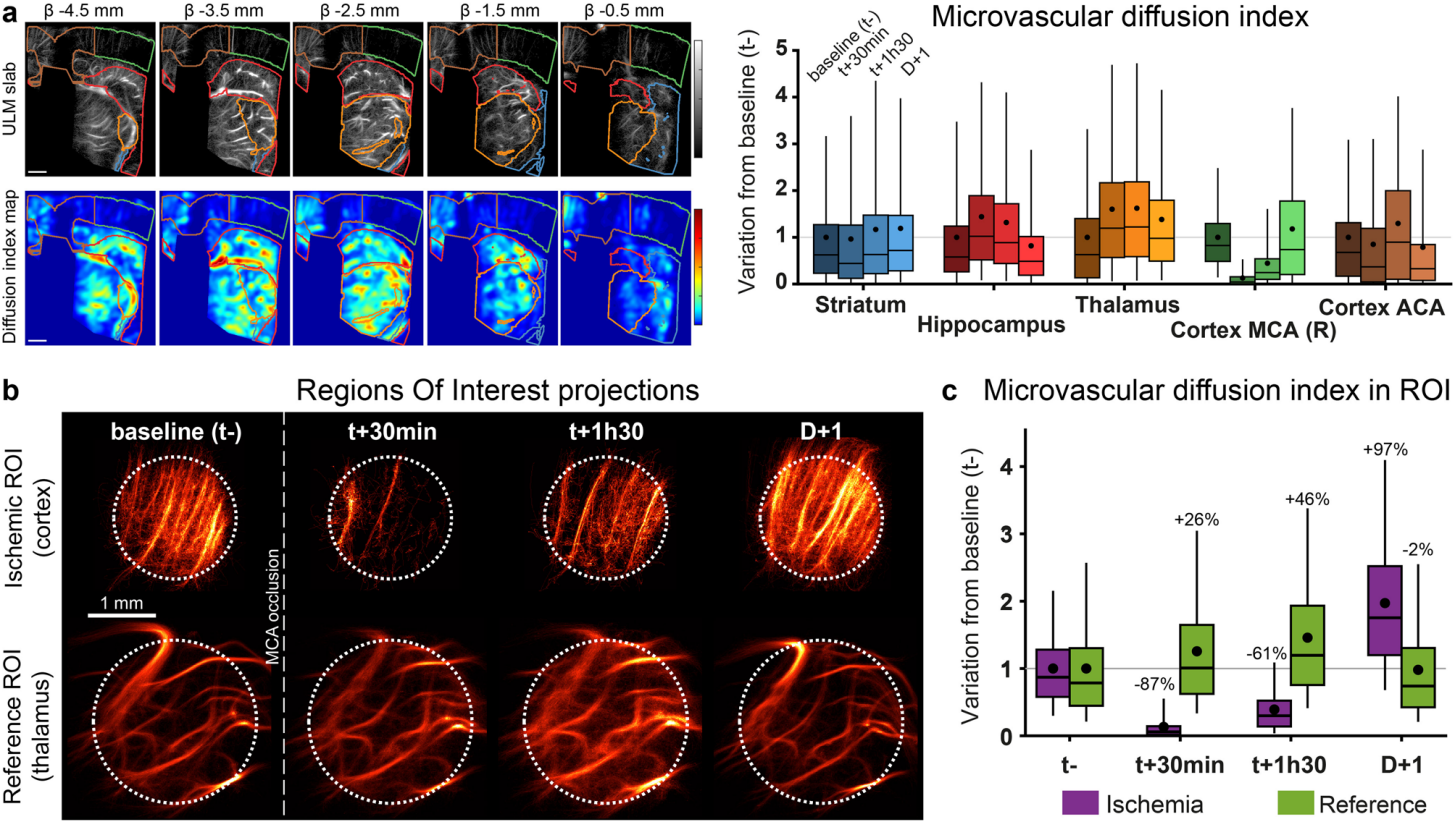
Sudden and massive loss of perfusion in the cortex. **(a)** Coronal slices of MDI with anatomical regions. MDI values inside each region normalized with t−. Scale bar 1 mm, slices of 600 µm. (n = 1). (**b)** Projection of the selected ROI in the cortex and thalamus as a reference. **c** MDI values for the ROI inside ischemia and the reference, normalized with baseline (t−). (Results of the analysis of 1 animal over 4.) Box plot: mean (dot), median (line), 1 and 3 quartile (box), 5 and 95% (whiskers). Figures were created with **(a–c).** MatLab 2019a (https://www.mathworks.com/products/matlab).

The MDI was computed for each anatomical region without prior localization of the ischemia. The hypoperfused region follows the cortical area until the central part of the cortex, perfused by the ACA (Fig. 4a). The MDI values were normalized in relation to the initial values for each anatomical region. All average MDI remained constant with similar standard deviations (SD) (Fig. 4a) except for the right cortex associated with the occluded MCA (Cortex MCA (R)), which underwent a massive loss down to 0.1 (SD 0.2) at 30 min. Later, this value increased up to 0.5 (SD 0.6) at 1h30 and confirmed the visual observation of the reperfusion. The day after, the value was restored close to the initial value at 1.2 (SD 1.3). As a comparison, in the neighbor’s regions, hippocampus, thalamus, and cortex ACA regions, the MDI drop was limited to 21% for the cortex ACA at 24 h compared to the baseline (t−). (MDI slices in Supplementary Fig. S1–2).

The unsupervised MDI can be biased by the placement of the probe on the skull’s heterogeneities. For that reason, the analysis was pursued by selecting particular regions of interest in the cortex and the thalamus as a reference. In the reference region, the vascularization is constant at all timepoints with minor variations. The extracted ROI presents a sudden lack of microbubbles in the cortex just after the MCA occlusion (Fig. 4b). The day after, the microvascularization returns to baseline with a dense population of trajectories and was confirmed by the MDI (Fig. 4c). For the reference region, the average value remained stable with a maximal value of 1.5 (SD 0.9) at t + 1h30. The MDI was more affected in the ischemic region with a drop of 87% (0.1 SD 0.2) at t + 30 min. The early reperfusion of the cortex raises the MDI to 0.4 (SD 0.4) at 1h30. The low standard deviation indicates a homogenous density of restored microvessels. The day after, the MDI overshot the baseline with a value of 2 (SD 1).

### Hemorrhagic stroke model

**Figure 5:**
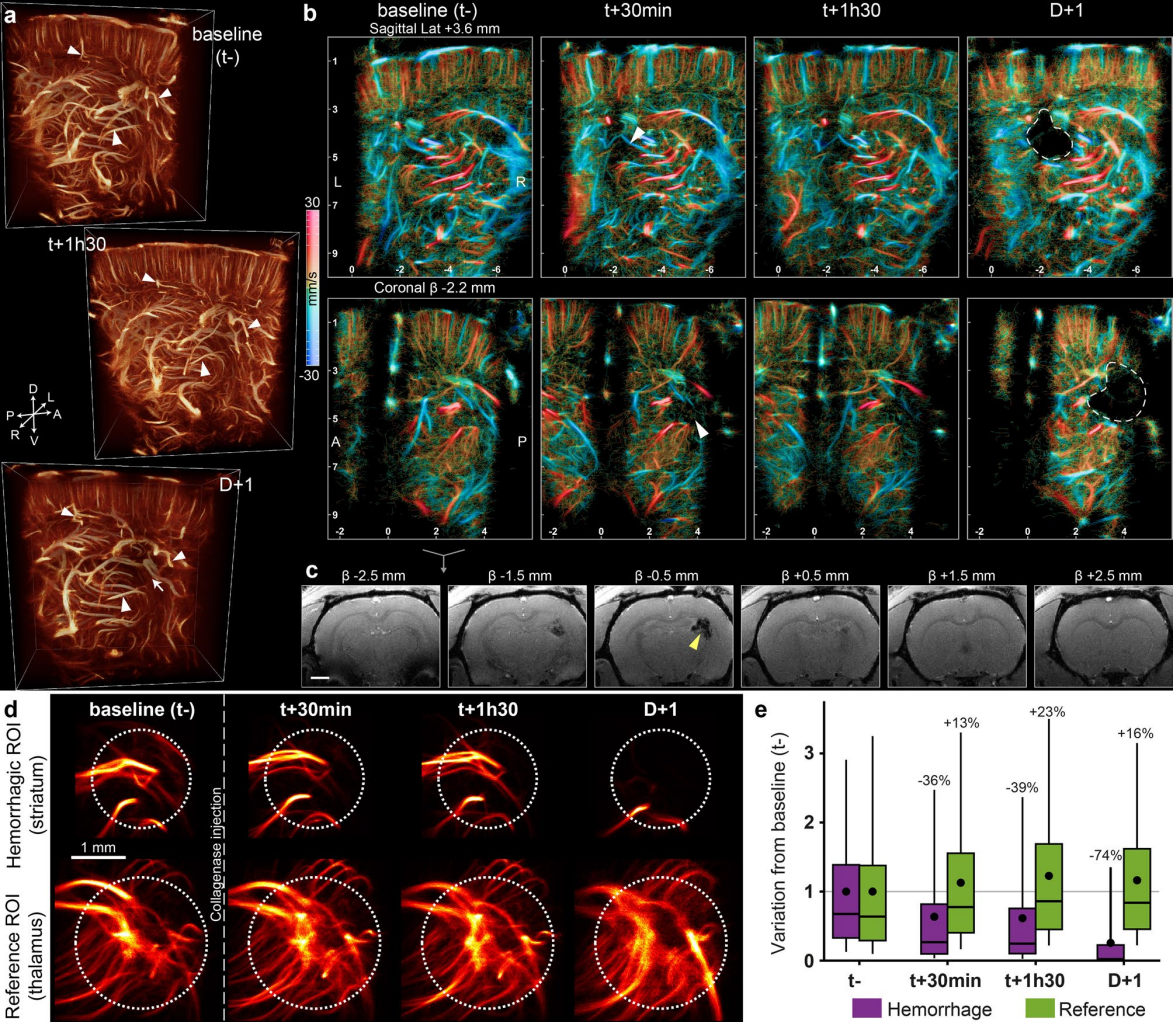
Collagenase induced hemorrhage in the striatum observed with 3DULM in the early phase. **(a)** 3D renderings before, and 1h30 after stroke’s induction, and the day after (Supplementary Video S2). (**b)** Sagittal and coronal slices of 600 µm with velocity rendering at different timepoints. Hemorrhagic core wrapped with a white dotted line. ${V}_{max}=30\mathrm{ mm}/\mathrm{s}$. Upward flow: red, downward flows: blue. (**c)** T2* MRI at D + 1. Scale bar: 2 mm. Yellow arrow targets the hemorrhagic core. (**d)** Projection of the selected ROI in the cortex and thalamus as a reference. (**e)** MDI values for the ROI inside the hemorrhagic core and the reference normalized with the baseline. (Results of the analysis of 1 animal over 5.) Figures were created with (**a**) Amira 2019.4 (https://www.thermofisher.com/amira-avizo), (**b–e**) MatLab 2019a (https://www.mathworks.com/products/matlab).

The hemorrhage model was imaged at different timepoints after onset. The round shape of the hemorrhagic core can be identified in the volume rendering at D + 1 (white arrows) (Fig. 5a, Supplementary Video S2) surrounded by large blood vessels present at all timepoints (white triangles).

Within the first hours, the lack of perfusion occurs mainly in small vessels, with a loss of microbubbles trajectories mainly visible in the sagittal slices at t + 30 min and t + 1h30 (white arrows) (Fig. 5b). The shaded and diffused mesh of trajectories disappeared gradually the first day after onset and left a larger dark zone the day after.

The hemorrhage location and volume were validated with a T2* MRI sequence at 24 h post-onset (Fig. 5c). A rounded shape appears hypointense on few slices with a 7 mm^3^ lesion.

The extraction of ROIs shows a constant and similar blood vessel organization in the thalamus as a reference for normalization (Fig. 5d). The hemorrhagic ROI shows a progressive loss of small vessels in the first hours. At t + 30 min and t + 1h30, the largest vessels are almost stable, but the complex mesh with a low intensity disappears. This type of trajectory can be related to the capillary mesh. The day after stroke onset, the tissue necrosis visible on MRI (Fig. 5c) hinders the vascularization of any vessels (Fig. 5d).

The MDI analysis confirms the stability of the reference region (Fig. 5e) with a maximal variation of 23% of the baseline at 1h30 (1.23 SD 1). On the other side, the hemorrhagic ROI decreased gradually with a loss lower than 35% the first day (0.6 SD 0.9 at 30 min and 0.6 SD 0.9 at 1h30), and 74% the day after (0.3 SD 0.6). The high standard deviations in the first two hours reflect areas with high MDI indices related to large vessels combined with underperfused regions where the capillary bed is missing (Supplementary Fig. S1–2).

### Multiple animals’ analysis

**Figure 6:**
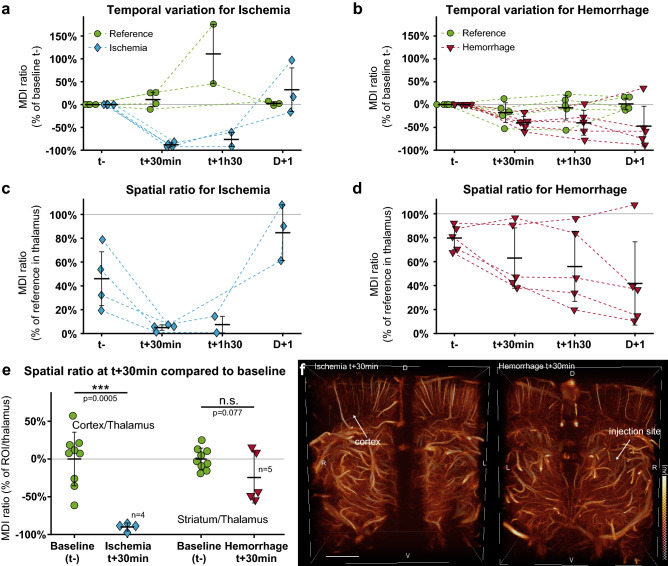
Comparison between ischemic and hemorrhagic models. Variation of the MDI normalized prior onset (t−) for each ROI in the ischemic (**a**) and hemorrhagic (**b**) stroke and reference ROIs. Ratios between the ischemic (**c**) and hemorrhagic (**d**) strokes ROI and the reference region. (**e**) Normalized MDI ratio of the lesion site and the reference region, compared to the baseline (t-). All animals were pooled for baseline ratio in cortex and striatum (n = 9). Student t-test with equal variance (**f**) Volume renderings of ischemic (left) and hemorrhagic (right) strokes 30 min after onset. Scale bar 2 mm. Figures were created with (**a–e**) MatLab 2019a (https://www.mathworks.com/products/matlab), (**f**) Amira 2019.4 (https://www.thermofisher.com/amira-avizo).

The supervised analysis was repeated on several animals: n = 4 for ischemia and n = 5 for hemorrhage. All reference ROIs were located inside the thalamus, under the ischemic cortex and away from the collagenase injection site. For the hemorrhagic model, the ROI was adapted for each animal inside the center of the lesion. In the ischemic stroke model (Fig. 6a), all animals underwent a brutal and important loss of MDI the cortex ROI −88% (SD 4%) at 30 min respect to the baseline (t−). This massive loss correlates with the vascular territory downstream of the occluded MCA. The day after, most clots are no longer efficient, and all MCA have been reperfused (+ 32% SD 48%). In hemorrhagic models (Fig. 6b), the average MDI loss at 30 min is −40% (SD 14%) and drops to -47% compared to the baseline the day after with an extended standard deviation of 44%.

In the clinical case, the normalization cannot be done respected to the timepoint prior onset. The MDI of the stroke ROIs were then compared to the reference region. For ischemic stroke (Fig. 6c), the MDI in the ischemic cortex is lower than 10% of MDI value in the thalamus region during the first hours of the stroke (t + 30 min: 5% SD 2%, t + 1h30: 7% SD 7%), which is lower than the MDI ratio of the baseline (45% SD 23%). The day after, the MDI ratio increased respected to t- with a mean value of 85% (SD 23%). In the hemorrhagic model (Fig. 6d), only a slight tendency appears in several animals with a ratio lower than 50% with respect to the reference region with a progressive decrease (t + 30 min: 43% SD 4%, t + 1h30: 33% SD 11%), compared to the initial ratio of 80% (SD 9%). Finally, we normalized MDI ratios with respect to the baseline images and compared at t + 30 min (Fig. 6e). The reference value was devised from all animals prior to stroke induction (n = 9). For ischemic stroke, the damaged region in the cortex presents a loss of 90% of the baseline (SD 5%, p = 0.0005, n = 4). In the induced hemorrhagic lesion, MDI losses were more variable due to the individual response of each animal to our stroke model. In most animals, volume renderings could not identify lesion at the early stage (Fig. 6f). The overall cohort did not reach a significant level (p = 0.077, n = 5).

## Discussion

Ischemic and hemorrhagic stroke management in the acute phase of the disease relies heavily on neuroimaging to identify, classify, treat and monitor lesions. However, current neuroimaging solutions are incompatible with personalized medicine because over-booked by other pathologies, too expensive, and cannot be used bedside. Compact and portable ultrasound imaging devices are emerging, but the sensitivity and resolution of transcranial exploration remain insufficient for most tasks. ULM could partly solve those issues by combining large field-of-views with acceptable resolution and sensitivity to the cerebral blood flows.

In this study, ULM allows imaging microvascularization patterns of ischemic and hemorrhagic strokes. Besides the obvious differences in injection sites, the aspects and timing of the vascular alterations were also drastically different. Ischemia appeared as a well-defined vascular territory with a major loss and no temporal variations within the first hours (Fig. 4). Hemorrhages appeared with a more diffused loss worsening gradually over time (Fig. 5).

The ischemic lesion is clearly highlighted by ULM during the first hours after onset. A large and well-delimited area in the brain appeared completely non-perfused immediately after inducing the arterial occlusion. This area also matched the perfusion territory of the MCA (Fig. 3). It also fits with the lesion site as seen on MRI at 24 h, which is in accordance with previous studies^46^. Some variations in perfusion can be observed in separated regions, although of a smaller extent and without causing any lesion. In the cerebral hemorrhagic rat model, we observed the presence of diffused hypoperfused regions in the hyperacute stage (Fig. 5). This diffused aspect was highlighted by the high standard deviation of the MDI (Fig. 5e) compared tighter value in the ischemic lesion (Fig. 4c). These regions did not match any anatomical structures with round or elongated shapes. It appeared to affect small vessels gradually in the early stages and a maximal loss the day after the hemorrhage induction. In the rats with the smaller hemorrhagic lesions, the hypoperfused region was too small to be detected by ULM, and the MDI remained constant, whereas the MRI confirmed the hemorrhage.

For the diagnosis of stroke, the spatial comparison of MDI provides a characterization without prior imaging. By normalization of the MDI with a known and healthy region, the thalamus, cortical ischemic strokes underwent a drop of more than 85% for all animals within the two first hours (Fig. 6). After normalization with baseline images, the comparison at t + 30 min reveals an efficient detection of ischemic stroke (P = 0.0005) in the cortex (Fig. 6). The good sensitivity rises from the high sensibility to moving flows with ULM and the total occlusion of the blood flow. Furthermore, in the early phase of hemorrhagic stroke, the blood circulation is less impacted, and no significant losses were observed with ULM. The hemorrhagic mechanisms first involve the denaturation of hemoglobin with the evolution of these products. They can be successively identified with CTscan or MRI^4^. Later, as a result of hypertension, the necrosis region can appear totally under perfused on ULM imaging with any microbubbles detected (Fig. 5c at t + 24 h).

In all animals, the ULM processing reveals a fine description of the microvascularization. In most situations, the lesion could only be partially observed. Indeed, large regions under the sagittal sutures and crests of the skull appeared shadowed. These complex and heterogeneous structures induced aberration and attenuation, which hindered ultrasound imaging. By tilting the probe, shadowed regions can be projected to visualise vascularization under crests (Supplementary Fig. S3). The volumetric property of this system, compared to restrict 2D ultrasound imaging^24^, facilitates the observation and analysis of microvascularization with no user dependency and optimal placements over the skull. Besides these limitations, resolution and sensitivity were^30^ 30 µm for the smallest detectable vessel confirmed with several microbubbles, and a sensitivity up to 73 mm s^−1^. Smaller vessels and capillaries were not resolved on the images, although they still appear like a complex and unresolved mesh. Moreover, we have also shown that the slowest microbubbles are affected by the clutter filter process^30^ and are most likely unobservable with the current technique of acquisition and filtering.

Most quantitative indices built from ULM and contrast-enhanced data, in general, suffer from a lack of normalization. Indeed, although injection doses and animal vitals were kept as constant as possible, the circulating microbubbles concentration still varies significantly and affects the accuracy of biomarkers based on intensity. This variability can be explained by the initial concentration of Sonovue^47^, the quality of the intraveinous injection, the attenuation of the skull, and the signal-to-noise ratio inside the brain. Thresholds can also be applied to images of vascularization to highlight vessels and get a quantification of the cerebral blood volume.

For microvascular imaging, ULM is mainly sensitive to flow perturbations with drastic reductions in flows. Both detection and tracking of microbubbles focus on flowing particles with a velocity range of 4.3 to 28.4 mm/s^30^ and highlight arterioles and venules. At this scale, the resolution and sensitivity of the system provide a good detection of vascular issues, but it fails at the capillary scale. In the context of stroke diagnosis, rapid and important microvascular losses are characteristics of a heavy ischemic stroke in the early phase with large underperfused regions. Conversely, early hemorrhagic strokes do not present totally underperfused areas and could not be identified with ULM within the first hours. The results of MDI at t + 24 h after onset were not relevant for ischemic stroke detection because of recanalization and could be associated with an old hemorrhage (Fig. 6e). The pattern of hypoperfusion in the hemorrhagic lesion at t + 24 h draws similarities with recent ischemia (Figs. 3b, 5b). However, in a clinical context, the time to onset is estimated by the symptoms and must exclude either old ischemic or hemorrhagic lesions. Therefore, with the descriptions of symptoms and an estimated time to onset, ULM in the early phase may discriminate the subtype of stroke: an MDI loss of more than 90% compared to the baseline can be associated with ischemia; otherwise, the stroke can be classified as hemorrhagic (Fig. 6e).

The velocity and direction of microbubbles were also investigated but no significant outcomes were found between ischemic and hemorrhagic strokes (Supplementary Fig. S5–6). In the analysis, the modifications of mean velocity or variance were mainly related to a smaller population of microbubbles, for example, in the ischemic model.

This study can be seen as a first proof of concept toward transcranial human ULM. It has already been validated in 2D in the brain^24^ and various abdominal organs^48^ by reducing the frequency to 2–3 MHz. In the brain, the increased field-of-view will resolve numerous anatomical regions with an isotropic resolution. The temporal window offers a privileged ultrasonic window with an absorption equivalent to those involved in this study (−18 dB at 7.8 MHz). Aberration and attenuation issues induced by the skull have been explored in the field of transcranial Doppler^49^ and recently for 2DULM in humans^24^. Its generalization to 3D will create further challenges induced by diffraction, and clinical translation is well within the scope of current ultrasound advances. Besides, the placement of the probe on this temporal window gives direct access to the main cerebral arteries^18^, their associated vascular territories, and particularly MCA territories that represent half of the cerebral ischemic strokes^50^. However, the present extended field of view used in this study is insufficient to observe the whole brain, thus limiting the possibility of revealing some stroke events depending on their location. Further developments would be needed prior to translation to the clinic.

This new imaging technique focuses on the dynamic vascular flow while MRI and CTscan are more sensitive to cellular and tissular changes induced by stroke mechanisms. Meanwhile, optical-based imaging techniques are limited by the skull and absorption. Cerebral ischemic strokes are mainly represented by sudden and total vascular occlusion and are particularly visible when imaged in the first hours after the onset. The ULM investigations combined with estimation of the time to onset reveal the presence of a hypoperfused area with a precise definition. With high dissemination and availability, this technique could bring a first characterization of massive ischemic stroke that represents 1 stroke over 5, with the possibility to be performed bedside, at the bed of patients or in the ambulance. A recent study with 2D functional ultrasound has yielded the prediction of the lesion size in ischemic stroke^46^. The administration of thrombolytic agents reduces the lesion size and must be injected as early as possible. With this study, we expect an early diagnosis of ischemic stroke versus hemorrhagic one in order to reduce the time to needle for fibrinolysis + /− thrombectomy and wider the number of eligible patients, and finally reduce long-term sequelae. It could also find an application in monitoring the recanalization of arteries after a therapeutic action, for example, a mechanical thrombectomy of an ischemic stroke.

Volumetric ULM is an emerging imaging technique that provides high resolution and sensitivity for brain vascular imaging with a simple and safe system, even through the skull of the rat. The sensitivity to intravascular moving microbubbles highlights induced ischemic strokes in rats and allows us the discrimination between ischemic and hemorrhagic strokes in the early phases. Its extension to human imaging could support current neuroimaging systems in stroke management, especially for very early detection of cerebral ischemia in the ambulance.

## Supplementary Information

Supplementary Information.

Supplementary Video S1.

Supplementary Video S2.

## Data Availability

The datasets used and analysed during the current study are available from the corresponding author on reasonable request.
