# Publisher Correction: 3D transcranial ultrasound localization microscopy for discrimination between ischemic and hemorrhagic stroke in early phase

**DOI:** 10.1038/s41598-022-21967-x

**Published:** 2022-10-19

**Authors:** Arthur Chavignon, Vincent Hingot, Cyrille Orset, Denis Vivien, Olivier Couture

**Affiliations:** 1 Sorbonne Université, UMR 7371 CNRS, Inserm U1146, Laboratoire d’Imagerie Biomédicale, 15 Rue de l’Ecole de Médecine, 75006 Paris, France; 2 UNICAEN, Inserm U1237, Etablissement Français du Sang, Physiopathology and Imaging of Neurological Disorders (PhIND), GIP Cyceron, Institut Blood and Brain @ Caen-Normandie (BB@C), Normandie University, Caen, France; 3 Department of Clinical Research, Caen-Normandie University Hospital, CHU Caen, Avenue de la Côte de Nacre, Caen, France

Correction to: *Scientific reports* 10.1038/s41598-022-18025-x, published online 26 August 2022

The Supplementary Video S1 and Supplementary Video S2 files published with this Article contained errors, where some of the descriptive labels of the video were omitted.

These errors have now been corrected in the Supplementary Video S1 and Supplementary Video S2 files that accompany the original Article.

