# Supplementary material for: 3D transcranial ultrasound localization microscopy for discrimination between ischemic and hemorrhagic stroke in early phase
Source: Sci Rep. 2022 Aug 26;12:14607. doi: 10.1038/s41598-022-18025-x (PMC9418177; doi:10.1038/s41598-022-18025-x)
Supplement: Supplementary file 1 — Supplementary Information. [file 41598_2022_18025_MOESM1_ESM.pdf]

Supplementary materials

A. 3D Ultrasound Imaging

The 1024 ultrasonic matrix probe with 300 µm element's pitch (central frequency 7.8 MHz, 56% bandwidth at -6 dB) (Vermon, Tours, France) was driven by the unique ultrasound scanner Vantage 256 (Verasonics, Kirkland, USA) via a 4-1 multiplexer (Verasonics). Elements of the probe were placed on a 32 x 35 grid size, with three empty rows: 9, 17, 25, for an effective field of view of 9.6 mm x 10.5 mm. The 1024 channels of the probe were divided into four synthetic apertures representing four panels of 32 x 8 elements that can be connected to the 256 channels of the ultrasound scanner. By changing the state of the multiplexer, the ultrasonic scanner controls one of the four apertures. The switching time allows changing the state between the emission and the reception. Finally, to insonify and receive all signals from scatterers facing the probe with one plane wave, 10 transmissions were required with different couples of emission reception apertures. Transmitting and receiving apertures were used in the following order: (TxAp, RxAp) = {(1, 1), (2, 1), (1, 2), (2, 2), (3, 2), (2, 3), (3, 3), (4, 3), (3, 4), (4, 4)}.

The probe was held on with a custom probe holder fixed on the stereotaxic instrument holder. The skull was covered with echographic gel to match the impedance with the ultrasonic probe.

Volumes were obtained by compounding 5 tilted plane waves after a Delay-and-sum beamforming algorithm. Plane waves were sent in that order (Azimuth, Elevation) = {(0, 0), (-5, 0), (5, 0), (0, -5), (0, 5)} in degrees. 50 transmissions were required for one compounded volume. The pulse repetition frequency was set to 13 kHz for a 16 mm acquisition depth with a compounded volume rate of 250 Hz.

For each 3D ULM volume, 500 blocks of 200 volumes were acquired in 7 minutes at 250Hz compounded volume rate (CVR), with 256GB of raw data signal.

Excitation pulses were sent at 7.8 MHz, with 4 half-cycle and 16 volts (peak negative pressure 662 kPa, MI=0.24). The PNP was also measured behind a rat skull ex vivo, and the pressures did not exceed 400 kPa (MI=0.16), assuring no microbubbles destruction inside the brain.

Echoes were sampled a 31.2 MHz, giving 4 samples per period. Only the two first samples were kept every period to ensure accurate demodulation. 256 samples were necessary to reconstruct the volume with a depth of 11.4 mm. The reconstruction depth started at 3.7mm from the probe due to a limitation of the commutation time of the switch and to avoid near-field artefacts.

The 3D imaging did not require motion correction as the skull was restrained in a stereotaxic frame. No aberration correction was used for transcranial ultrasound imaging.

B. Microvascular Diffusion Index maps

MDI maps were computed for each animal, and the analysis was made on a particular region of interest inside the lesion (white dotted circle).

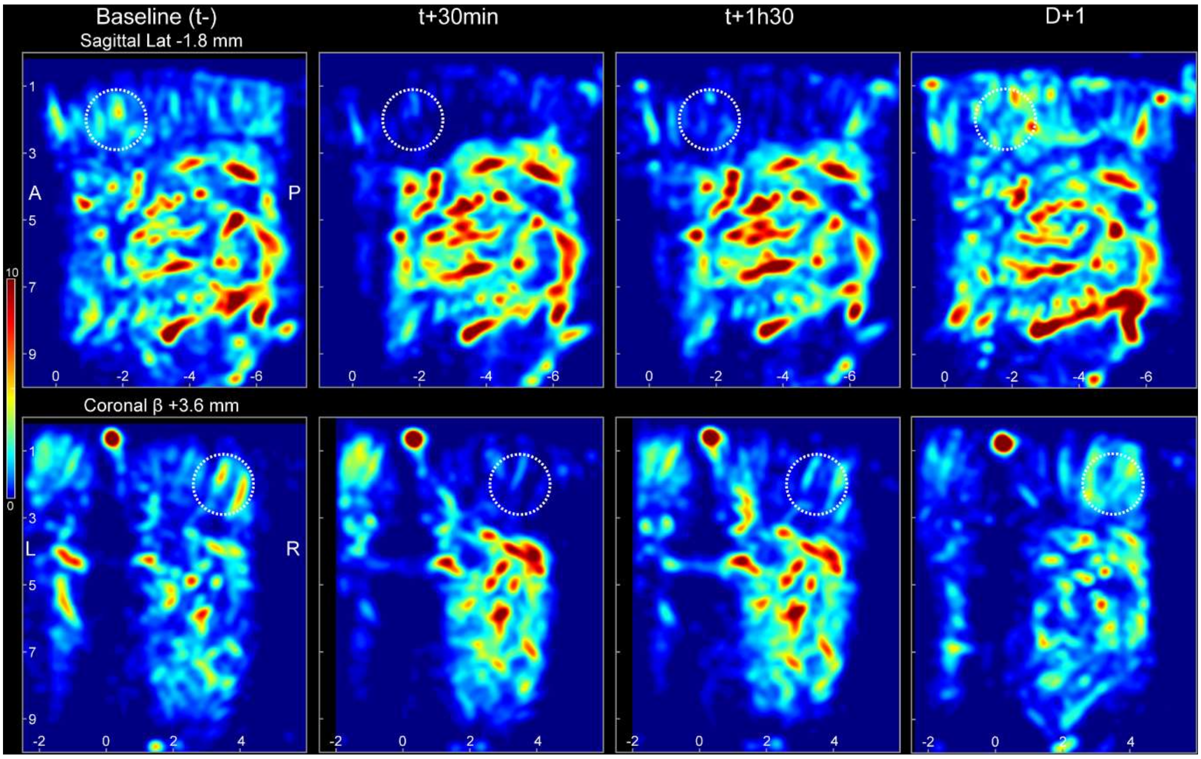

Sup. Fig. 1 Coronal and sagittal MDI maps of the rat brain before ischemic model induction, at 30min and 1h30 and the day after. (Created with MatLab 2019a, www.mathworks.com/products/matlab.)

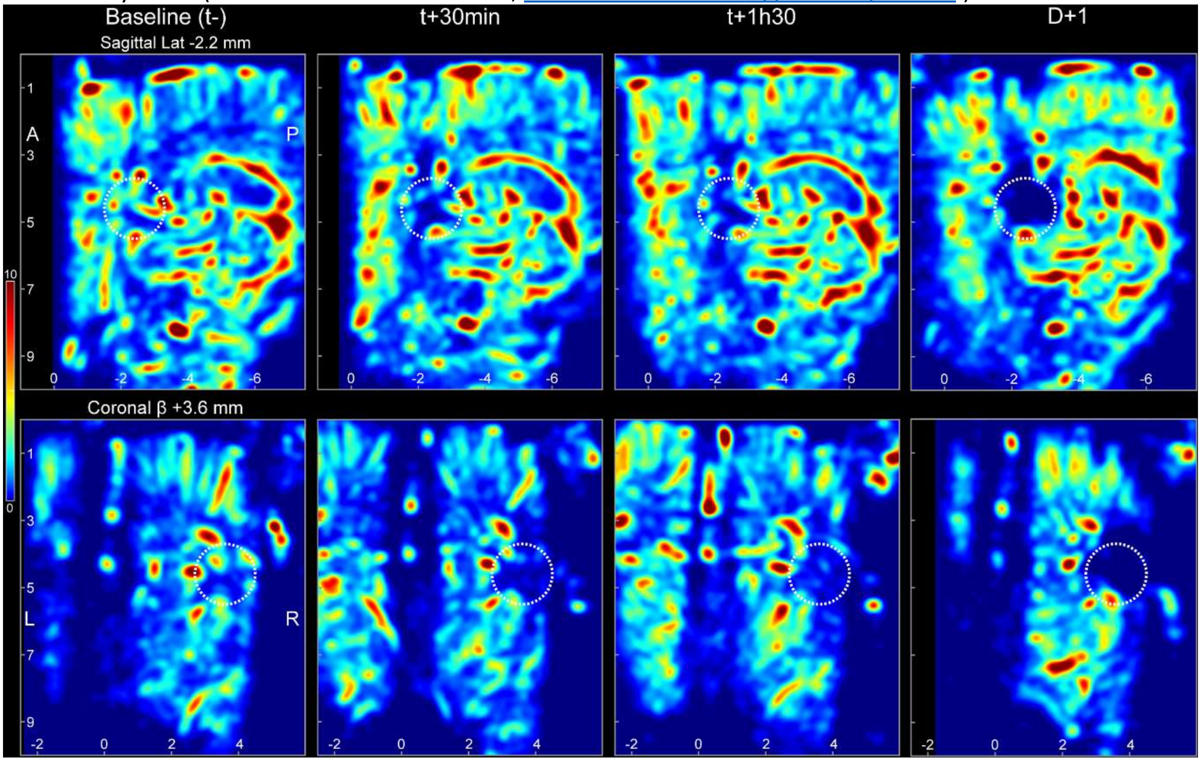

Sup. Fig. 2 Coronal and sagittal MDI maps of the rat brain before hemorrhagic model induction, at 30min and 1h30 and the day after. (Created with MatLab 2019a, www.mathworks.com/products/matlab.)

C. Toward ULM under crests and sagittal suture

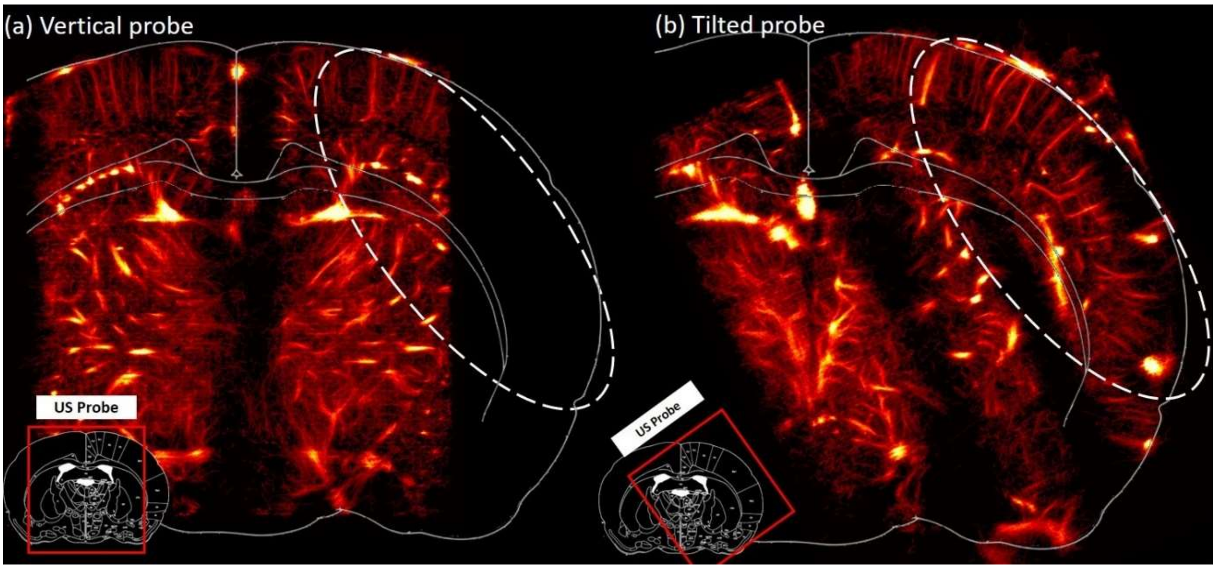

Sup. Fig. 3 Slices of 3D transcranial ULM of the rat brain with a vertical probe (a), and by tilting the probe (b) to focuses on cortex of the right hemisphere, under crests.
(Created with MatLab 2019a, www.mathworks.com/products/matlab.)

D. Lesions measurements

Lesions were measured with MRI at t+24h with ImageJ. Hemorrhagic lesions were extracted from T2* sequence and ischemic lesions with a T2 weighted sequence.

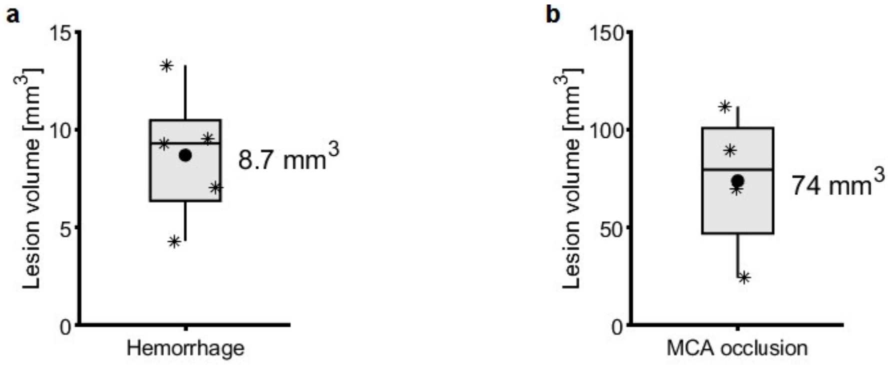

Sup. Fig. 4 Cerebral lesion at t+24h measured on MRI. **a** Hemorrhagic lesion, intracranial bleeding, measured on T2* sequence. **b** Ischemic lesion, oedema, measured on T2 weighted sequence.
(Created with MatLab 2019a, www.mathworks.com/products/matlab.)

E. Velocity and direction maps

The velocity magnitude and azimuthal directions of microbubbles' trajectories were plotted on a polar graph. For each region of interest (ischemia, hemorrhage and thalamus), all microbubbles entering penetrating the sphere were assigned a mean velocity and direction and compared along the different timepoints.

The eigenvectors of the principal component analysis are displayed as Eigen 1 and 2.

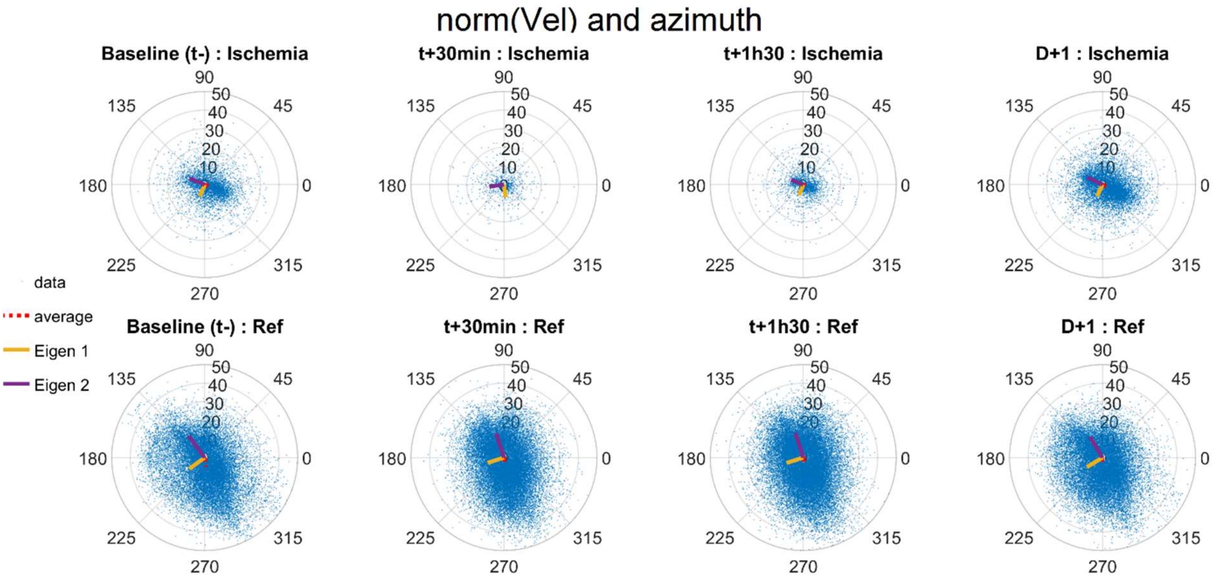

Sup. Fig. 5 Velocity distribution of microbubbles trajectories in the ROI for the ischemic stroke model before induction (t-), at 30min and 1h30 after, and the day after. (Created with MatLab 2019a, www.mathworks.com/products/matlab.)

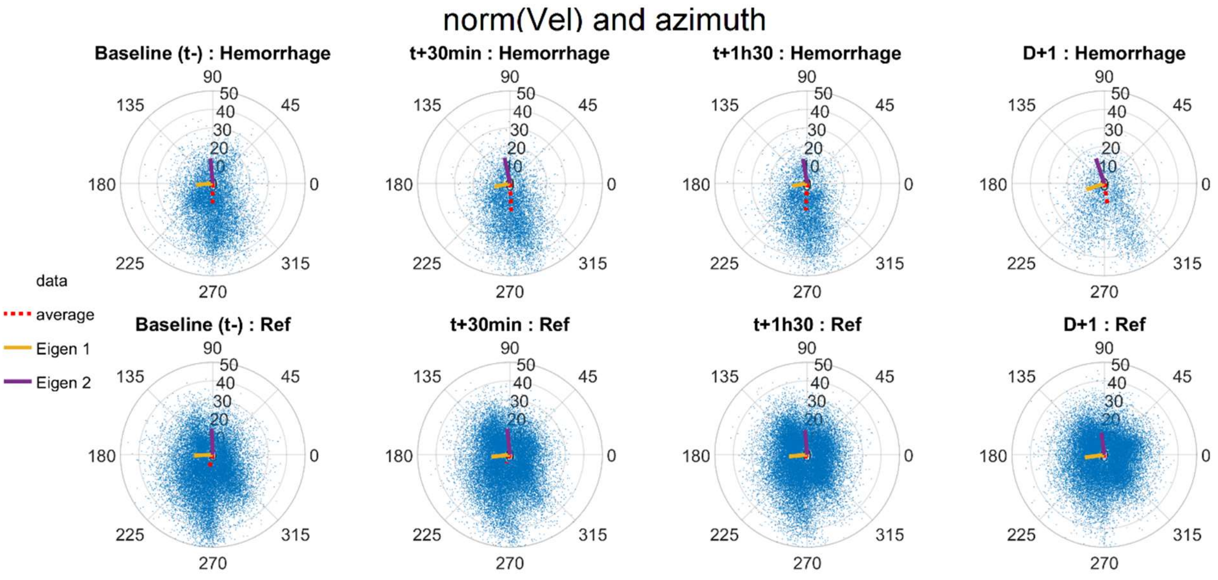

Sup. Fig. 6 Velocity distribution of microbubbles trajectories in the ROI for the hemorrhagic stroke model before induction (t-), at 30min and 1h30 after, and the day after. (Created with MatLab 2019a, www.mathworks.com/products/matlab.)
